# Marriage, bridewealth and power: critical reflections on women's autonomy across settings in Africa

**DOI:** 10.1017/ehs.2022.27

**Published:** 2022-07-06

**Authors:** Constance Awinpoka Akurugu, Isaac Dery, Paul Bata Domanban

**Affiliations:** 1 Faculty of Public Policy and Governance, SDD University of Business and Integrated Development Studies, PO Box WA64, Wa, Ghana; 2 Faculty of Integrated Development Studies, SDD University of Business and Integrated Development Studies, PO Box WA64, Wa, Ghana

**Keywords:** Bridewealth and brideservice, women's autonomy, sexual conflict theory mystical forces, women's subordination, Africa

## Abstract

This article examines ongoing discourses on the importance of the marriage payment and its role in constraining women's autonomy across societies in Africa. First, we review how bridewealth has been conceptualised across multiple disciplines, including the work of evolutionary human scientists. We then summarise our research grounded in residential ethnographic fieldwork data collected over a period of a year in a rural settlement in north-western Ghana. Feminist accounts on women's lived experiences throughout bridewealth practising societies point to their subordination. In some contexts, including northern Ghana, bridewealth is perceived as engendering women's oppression. To liberate women from patriarchal norms, some gender advocates call for undoing of the institution of the marriage payment. Nonetheless, the women who bear the brunt of gendered oppression and the men who derive patriarchal dividends from it are averse to this undoing discourse as the bridewealth normatively secures legitimacy for women. Undoing bridewealth may mean further rendering precarious women's status in the marital family. We conclude that rather than undoing the revered institution of bridewealth, there is need to build on culturally appropriate notions of communitarianism as encapsulated by the Ubuntu philosophy and indigenous systems such as the traditional courts for negotiating the rights of women.

## Graphical abstract

**Figure:**
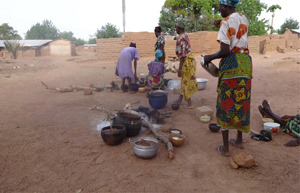

**Social media summary**: Bridewealth is perceived as the basis of women's oppression and normalised violence, complicating gender equality and women's empowerment.

## Introduction

How does the payment of bridewealth sculpt women's identities and their social positioning in patrilineal and patriarchal societies? What is the role of the marriage payment in negotiating legitimacy for woman in marriage? Does the connubial payment oppress women and constrict their autonomy? Might undoing the institution of bridewealth liberate women from the shackles of male dominance and oppression or could it worsen women's already tenuous social positioning within patriarchal societies? These are important questions that require analytical and strong theoretical attention in order to understand the implications of bridewealth practices for women's autonomy and their exercise of agency in societies in Africa. Bridewealth, sometimes used interchangeably with marriage payment or brideprice, may be described as prestations, gifts/goods and services that are transferred from the groom's kin to the bride's family (Goody & Tambiah, 1973; Tambiah, 1989). In this perspective piece we draw on evolutionary perspectives, including sexual conflict theory, the marriage market and honest signalling to examine the practice of bridewealth and its role in solidifying relationships among families and in securing legitimacy for women in heteronormative marriage across societies in Africa (see also Dolphyne, 1991; Shope, 2006; Akurugu et al., 2021). We also reflect on the role of bridewealth in constraining the autonomy of women as well as the complexities involved in attempting to undo the revered institution. We discuss the problems that may come with ending the practice of bridewealth, including the potential to further disempower women (Behrends, 2002; Dery, 2015; Akurugu et al., 2021). This is achieved by drawing on residential ethnographic research conducted in a rural settlement in north-western Ghana combined with in-depth interviews with community leaders and scholars across two ethnicities in northern Ghana, namely Frafra and Builsa. In the process, we bring into dialogue contemporary discourses on the politics and economics of the connubial payment across Africa more broadly and the possibilities that bridewealth may offer in imagining liberatory and progressive gender subjectivities. In particular, we draw on the works of Goody (1956, 1969), Borgerhoff Mulder (1995), Shope (2006), Anderson (2007), Apostolou (2008) and Horne et al. (2013) as foundational frameworks for our analysis. Our aim is to closely engage how these frameworks may nuance understanding of bridewealth transfers as strategic or adaptive behaviour, which may potentially have benefits for one or both genders under certain conditions.

Several anthropological studies, including works of evolutionary social scientists, document in detail the practice and determinants of the marriage payment across human societies (Murdock, 1967; Anderson, 2007; Apostolou, 2008). These studies draw attention to its pervasiveness. In Murdock's (1981) *Atlas of World Cultures*, a catalogue of cultural practices of societies where ethnographic data was then available, 226 out of 563 societies practised bridewealth. Bridewealth is the most common means through which a marriage becomes legitimate across most cultures in Africa. Customarily, bridewealth is practised in 90% of societies across Africa (Murdock, 1967). While bridewealth payment has been an enduring cultural practice over the years, it has equally survived neoliberal capitalist forces. Consequently, the marriage payment has seen modifications across contexts, as our case study will show (Shope, 2006; Anderson, 2007).

Although the basis of a legitimate marriage arguably brings dignity and respect to women and men and their offspring, the institution of bridewealth is frequently perceived and portrayed as the bane of women in most patrilineal societies (Horne et al., 2013). In particular, it is viewed as combining with virilocal residential arrangements to further disadvantage women. For the purpose of this perspective paper, a virilocal residential arrangement involves a situation whereby the married couple take up residence with or near the husband's family. In a male-centric society, such arrangements determine inheritance systems in ways that constrain and push women to the periphery of society. Indeed, the marriage payment is considered by scholars of international development and feminist writers as antithetical to gender equality and women's empowerment as it ostensibly erases women's voices and autonomy (see Wendo, 2004; Anderson, 2007). In view of its potential to disempower women, some scholars and gender activists have called for the abolition of the practice. Yet such a call has received mixed reactions. For example, ethnographic evidence has suggested that the key actors of the marriage payment abhor the undoing discourse precisely because of its potential to worsen women's ambivalent positioning in the marital family (Akurugu et al., 2021).

In contrast, we propose a need to build on indigenous cultural institutions and values which are widespread across Africa, including the Ubuntu philosophy as a site for negotiating gender equality and women's empowerment through culturally innovative ways. The rest of the article is organised as follows: in the ensuing section we examine discourses on marriage payment across Africa, in Ghana and northern Ghana to develop a framework for our analysis. This is followed by a presentation of our case study of bridewealth and women's autonomy in northern Ghana composed of the research context, methodology and key results. The case study primarily relies on ethnographic data gathered in a rural settlement in north-western Ghana and is complemented by in-depth interviews held with gatekeepers of two ethnic groups, also located in northern Ghana. The ethnicities include Builsa and Frafra in the north-eastern part of Ghana. The case study commences by introducing the research context, methodology and results. This is followed by a section on women's constrained autonomy and how previous efforts have attempted to resolve it. The final section reflects on the implications of our findings for discourses on marriage payment and women's autonomy across Africa.

## Sexual conflict theory

In evolutionary anthropology, sexual conflict theory refers to ‘conflict between the evolutionary interests of individuals of the two sexes’ (Parker, 1979: 124), that is, when the evolutionary interests of females and males differ, leading to sexually antagonistic selection, divergent selection that acts in a beneficent way to one sex and maleficent to the other. Sexual conflict ‘occurs when the optimal value of a trait differs between the two sexes’ (Borgerhoff Mulder & Rauch, 2009: 201). Sexual conflict theory is useful in this perspective research inasmuch as it offers insights for understanding the imbalances in men's and women's evolutionary interests in marriage and how one sex or the other might derive more net benefit or lose out. The theory has been instrumental in gaining deeper insights into sexual conflicts over mating and fertilisation among non-human primates in evolutionary studies. In studies involving humans, research reveals that women's strategies are often constrained by the forceful and manipulative behaviour of men and their kin and our analysis of marriage payment in northern Ghana below resonates with this finding. The role of men and their kinsmen in restricting women's autonomy looms large in marriage and this reflects the communitarian valence of the ethnicities under consideration in this study. Closely related to the gains and losses associated with the marriage payment as encapsulated by the sexual conflict theory is the concept of the marriage market developed by Gary Becker (1991). The marriage market is an analogy that draws heavily on economic principles and assumptions. The marriage market framework analyses the transfer of gifts upon marriage; both brides and grooms have various qualities that have great potential for promoting efficiency within the domestic sphere of the home and in the economy. The market rewards the mates appropriately, ensuring that brides and grooms gain in a commensurate manner in the mate matching.

### Marriage payment and women's autonomy in Africa

Across societies in Africa, the phenomenon of bridewealth is a central component of marriage. Bridewealth performs important and contradictory roles in securing yet complicating various kinds of rights and entitlements for the conjugal couples (Anderson, 2007; Horne et al., 2013; Posel & Rudwick, 2013). In most patrilineal societies, bridewealth secures dignity, recognition and respect for both the bride and her children in the new family (Shope, 2006; Anderson, 2007; Behrends, 2002; Akurugu et al., 2021). Nonetheless, the practice of bridewealth tends to deepen existing gender inequalities between men and women. For example, the practice draws on patriarchies to further oppress women as well as constraining their autonomy and agency, especially if combined with arranged marriage. Some scholars argue that bridewealth may even be a significant set-back to the achievement of gender equality and women's empowerment (Wendo, 2004; Shope, 2006; Horne et al., 2013; Dery, 2015; Akurugu et al., 2021).

In recent times, within the context of neoliberal market integration, dwindling natural resources and the monetisation of hitherto uncommercialised resources, the connubial payment has seen modifications, specifically in reduction in quantity across some contexts (Anderson, 2007; Akurugu et al., 2021). Yet in other contexts, notably urban societies across Africa, the connubial payment has soared. For instance, among the Zulu people of South Africa, Shope (2006) reports a change in payment patterns from cattle to cash as well as modifications in the practice. While acknowledging the significance of the *lobolo*, bridewealth, Shope noted that the amounts and items associated with the payment have soared over the period. Indeed, many scholars in South Africa have blamed the rising cost of *lobolo*, which tends to delay or even disincentivise marriage among young people (Posel et al., 2011; Posel & Rudwick, 2013). In the face of commercialisation, general integration into the market system and excessive focus on financial gains by the bride's kin and attacks by capitalism and Abrahamic religions, the institution of the bridewealth is enduring (Borgerhoff Mulder, 1995; Shope, 2006). For decades, anthropologists have studied bridewealth and its impact on family ties, productivity and social status. For their part, feminist scholars have been interested in the role of bridewealth in women's reproductive decisions, sexuality, ownership and control of productive resources (Shope, 2006; Horne et al., 2013; Akurugu, 2021). These studies point to the subtleties and the diversity that attend the payment.

Anderson (2007) and Apostolou (2008) offer very useful cross-cultural and historical analyses of bridewealth. Anderson (2007) provides important insights into the existence and extent of marriage payment across diverse socio-economic and historical settings and the transformations that various kinds of conjugal payments – bridewealth (prestations are transferred from the groom's kin to the bride's family), groomprice (payment is transferred from the bride's kin to the groom and his family) and dowry (payment is from the bride's family to her) – have undergone. Based on the cross-cultural analysis, the author notes that the amount of bridewealth in some societies is dependent on the rights transferred at marriage while in others it is constant irrespective of the income levels of the brideprice-paying family. In the case of our study, discussed below, the latter is applicable. In Kenya, for instance, Borgerhoff Mulder (1995) shows that the connubial payment depends on the circumstances of the bride, such as being divorced or the number of expected children or the groom's family background. Anderson also draws attention to the paucity of empirical data on this subject matter. The lack of relevant empirical data is reflected in some of the unsettling generalisations and stereotypes that accompany dominant debates on the marriage payment. For instance, Anderson writes: ‘The general pattern seems to be that brideprice exists more frequently in primitive, tribal, and often nomadic societies’ (2007, p. 155). However, the bridewealth practising ethnicities of northern Ghana are likely to be appalled by these unflattering labels. Our analysis departs from these kinds of one-sided statements by anchoring analysis on empirical data.

Anderson also considers economists’ perspectives on bridewealth, drawing on Gary Becker's (1991) marriage market framework described above. Becker deploys the marriage market as a metaphor to explain the processes involved in selecting a marital partner to optimise anticipated well-being. Across rural societies in northern and southern Ghana, the theory of the marriage market is relevant inasmuch as prospective brides and grooms participate in such social activities as funeral ceremonies and on occasion meet their marital partners at these functions. Yet within the context of rural northern Ghana, particularly at the time of Becker's work, the agnatic kin, the paternal relations, of both bride and groom played important roles in selecting mating partners. Indeed, the kinsmen and kinswomen are important stakeholders in sustaining the marriage and we suggest that this atomistic conception of the marital actors that characterises much of the discourses on bridewealth (Apostolou, 2008; Borgerhoff Mulder, 1995), attributing agency to the partners/parents only, risks eliding the important role of entire families and communities in the marriage. It is within the understanding of the important role of the extended family in the marriage that we propose the need to draw on the communitarian valence of the ethnicities in northern Ghana encapsulated by the Ubuntu philosophy to negotiate women's rights and eradicate marital violence (Dery & Bawa, 2019; Akurugu et al., 2021). As well, the commercialised notion of bridewealth leaves much to be desired as other dynamics such as familial relations are at play, sometimes relegating the economic factors to the background. Among others, Anderson concludes by calling attention to the need for contextually nuanced data to shed further light on the shifts occurring across Africa.

Apostolou (2008) focuses on the thorny question of parents choosing suitable mating partners for their daughters. The author provides useful insights into the practice of the marriage payment and highlights evolutionary anthropologists’ perspectives on bridewealth and brideservice as compensations for the lost labour of daughters and/or as ‘honest signalling’ of a son-in-law's wealth and capabilities, enabling parents to assess the suitability of the groom. A summary of explanations for the practice of the marriage payment, as the author discusses, includes compensating for the loss of the bride's labour, guaranteeing respectable treatment for the bride in the marital family and securing legitimacy for the bride and her children in the marital family (Murdock, 1949; Ogbu, 1978, see also Behrends, 2002; Akurugu et al., 2021). All of these are relevant to the context of our discussion. For example, while citing Zahavi's (1975) theory of honest signalling, Apostolu highlights how bridewealth and brideservice, a form of marriage transaction where services in the form of labour are rendered by the groom as payment for a bride, may serve as a function of testing a prospective son-in-law's suitability. According to Apostolu (2008), certain questions are asked in the process: does he have sufficient resources to support his future family? Is he fully committed to the marriage? Without the marriage payment, it may be challenging for parents to assess suitability in this manner. Thus, removing bridewealth could have detrimental impacts on women. Bridewealth represents an important means by which parents exercise control over the selection of grooms for brides. Similar to Anderson, Apostolou appears to downplay the role of the extended family in mate-matching. Within the context of rural societies in northern Ghana, normatively, the selection of suitable marital partners is a collective responsibility of the extended kin. In these settings, normative institutions rather than the parents are in charge of the bride and groom selection. The focus on the parents in this and many other studies is probably a reflection of the importance of the nuclear family in certain contexts. Yet in contexts where communitarian values are strongly upheld, as in the context of northern Ghana, mate selection is the task of many actors in the family. In the context of northern Ghana, both the bridewealth and brideservice are not executed single-handedly by the prospective groom, but instead by his agnatic kin and peers, respectively. As such, it is not clear to what extent marriage transfers can be said to honestly signal information about the commitment and economic potency of the suitor specifically, as opposed to providing information about his wider kin in many contexts across Africa.

Within the context of Africa, Borgerhoff Mulder's (1989, 1995) study of the Nilotic Kipsigis of Kenya examines bridewealth and women's constrained autonomy from the precolonial to the postcolonial era. The author argues that bridewealth in that setting is determined by women's perceived reproductive and labour value. Thus, early maturing brides attract higher bridewealth. In the past, women in marriage within Kipsigis societies had ‘secured rights’ and ‘considerable autonomy’ in using property that belonged to their husbands (Borgerhoff Mulder, 1995: 574). Yet the onslaught of colonialism and its oppressive patriarchies contributed to the dispossession of indigenous people of their land and the commercialised production of maize. While displaying African patriarchy, oppressive patriarchies of colonialism have worked in complex ways to empower men and disadvantaged African women. The payment among the Kipsigis is lower in the case of what Borgerhoff Mulder (1995: 567) terms ‘problem marriage’, which is a situation where the bride is pregnant or has already had a child prior to the payment. While Borgerhoff Mulder's results are compelling, the true extent to which bridewealth volume can be clearly linked to reproductive and labour value remains unclear. For instance, among the Frafra people, a wealthy woman may marry one or more women for her husband by providing the bridewealth. These women bear children in her name in the event of her being childless or to offer extra labour. This is similar to female husbands among the Igbo in south-eastern Nigeria (Amadiume, 1987). ‘Female husband’ is a term used by Ifi Amadiume to describe women who were wealthy in Igbo traditional societies and who acquired wives by paying the bridewealth of the women. This payment culturally made the wealthy women husbands to the brides. This woman-to-woman marriage secures social rights to the bride's children and service rather than sexual ones. Explaining the flexible gender system among the Igbo, Amadiume notes that the ‘female husband might give the wife a (male) husband somewhere else and adopt the role of mother to her but claim her services. The wives may also stay with her bearing children in her name’ (Amadiume, 1987: 42). By being able to control the services of other women, the female husbands accumulated more wealth and status, which further enhanced their social traction and authority vis-à-vis men. Yet the bridewealth is no less than in the case where the bride marries a normative male.

In the context of Ghana, Horne et al. (2013) examine restrictions that the marriage payment exerts on women's autonomy among the Ewe people of the Volta region. The study is revealing in that it draws attention to the limitations that ‘complete’ payment of bridewealth may exercise over women. Using an innovative vignette experiment, whereby participants were asked to consider marital scenarios wherein bridewealth had been not paid or had been partially or fully paid, the authors report that the more the marriage payment is nearing competition, the stronger the normative restrictions on women's reproductive autonomy and the weaker the restrictions on male violence. These authors conclude that the payment of bridewealth strengthens normative constraints on women's autonomy. However, while these results inform us about the consequences of the completeness of bridewealth payments within a specific setting where bridewealth is normative, it does not automatically follow that abolishing bridewealth as an institution itself will reap improvements in women's status. Answering this question requires a more nuanced investigation into how bridewealth as a normative institution interacts with and is embedded with patriarchal norms which subordinate women. Furthermore, the cultural significance of completing a bridewealth payment varies, and as we will argue, in some contexts rituals, rather than payments, may be more important in establishing when and how a women's status has been altered by marriage.

Specifically, in relation to northern Ghana, the findings of Dery and Bawa (2019) and Akurugu et al. (2021) offered sharp perspectives on the subject matter. Both studies are anchored on the important role of the marriage payment and its resilience amid neoliberal capitalism (see also Borgerhoff Mulder, 1995). The authors underscore the role of bridewealth as an institution that legitimates marriage. Marriage, and for that matter the payment of bridewealth, enables women in patrilineal contexts to gain new social status in society (Dery & Bawa, 2019). Against this backdrop, Akurugu et al. highlight the challenges of ill-informed gender advocacy that is premised on undoing the revered institution of the marriage payment, pointing out the dangers of such an enterprise. According to Akurugu et al. both the women in Dagaaba settings, the primary case study of our perspective piece, who bear the brunt of the marriage payment, including normalised violence and subordinate social positioning, and the men who derive patriarchal dividends such as control over women's productivity are opposed to undoing the institution of the bridewealth. This opposition is because the bridewealth secures legitimacy for both the woman in marriage and her children. Consequently, in its absence, the women risk losing the already tenuous position in the marital family. Yet the gender activists, mostly urban based educated women and clergymen, continue to advocate the undoing of this institution. Akurugu et al. conclude that women's rights advocacy should be sensitive to local norms and contextual dynamics in their efforts to ‘liberate’ the oppressed victims of patriarchal norms in northern Ghana. This article dialogues with and extends the argument offered by Akurugu et al. by incorporating the Ubuntu philosophy as an important means to draw on the communitarian features of African societies to negotiate women's rights and to undo marital violence in culturally appropriate manner.

The analysis so far highlights the diversity that characterises the practice of the connubial payment across settings in Africa. Bridewealth is an important practice, bringing respect, honour and legitimacy to the bride and groom and their kin. The exact amount of the marriage payment is contingent upon contextual factors. Yet the discourses seem to privilege the role of the parents and marital couples, eliding the complex network of relations that characterises marriage across rural settings in Africa. Most of the discourses, cross-cultural as they are, mean that contextual specificities are not always dealt with sufficiently. This article contributes to the literature in this field (Anderson, 2007) by bringing it together and engaging with ethnographic data in north-western Ghana in a way that enriches understanding of the practice of bridewealth and how it constrains women's autonomy. We argue that although the marriage payment may be perceived as the source of women's subordination, normalised violence, constrained autonomy and a setback to achieving gender equality, to undo the institution is to further worsen women's already tenuous social positioning in the marital family and to compromise on the legitimacy of the children in that union. In place of the colonising undoing, we turn to the communitarian philosophy of Ubuntu, prevalent across rural societies in Africa as an important space for negotiating women's rights and gender equality.

Ubuntu, an African humanist philosophy, highlights communitarianism – understanding the individual within the context of community – across African societies. According to Desmond Tutu, to say someone has Ubuntu is to acknowledge that ‘you are generous, you are hospitable, you are friendly and caring and compassionate. You share what you have. It is to say. “My humanity is caught up, is inextricably bound up in yours”’ (Tutu, 1999: 31). Ubuntu highlights the spirit of interdependence and incompleteness of the human person and this is encapsulated by the Zulu aphorism that: *Umuntu ngumuntu ngabantu*, a person is a person through other persons. The notion of a person becoming a person through other persons is replete across Ghanaian cultures including among the Dagaaba people, and this underscores the importance of interdependence. Ubuntu thus is about collectivity, being with others and seeing yourself as belonging to a larger society. This philosophy of interdependence enables African people to resolve social problems, including marital disputes by dealing with their problems in a positive manner, by relying on the inherent humanistic values that are embedded in African cosmology and forms of sociality (Nabudere, 2005). Ubuntu offers potential to build on pervasive strong communitarian values and solidarity that is inherent in most rural communities across Africa (Tamale, 2020) in order to resolve gender-based violence against women in ways that are culturally sensitive. By invoking the philosophy of Ubuntu, indigenous systems such as the traditional courts could be strengthened to appropriately resolve disputes, including marital ones without abolishing bridewealth.

## A case study of bridewealth and women's autonomy in northern Ghana

In this section we examine the case of bridewealth in northern Ghana, starting with the research context and methodology and then provide the results and discussion. We focus our analysis on an ethnographic research conducted in a Dagaaba settlement in rural north-western Ghana. This is supplemented by in-depth interviews conducted with members of two other ethnicities, also in northern Ghana, who have cultural affinities with the Dagaaba people to provide for comparison and to enrich our understanding of the marriage payment in this study context.

## The context and research methodology

This article relies primarily on data gathered during residential ethnographic field research from August 2013 to August 2014 in Serekpere, a rural settlement in north-western Ghana. The ethnographic fieldwork focused on gendered power relations. During the research, on a daily basis, the first author conducted a detailed participant observation and documented notes. The author was deeply immersed in the everyday realities and struggles of the research participants and this enabled her to gain in-depth knowledge of Dagaaba marriage practices. The ethnographic fieldwork was complemented by semi-structured interviews with both male and female cultural gatekeepers and individual women with in-depth knowledge of Dagaaba marriage practices and power relations.

At the time of the study Serekpere had a population of 1100. The settlement is 10 km away from the administrative capital of the Nadowli-Kaleo District, Nadowli, of the Upper West Region of Ghana. The ethnographic data is supported by data from five in-depth interviews with male and female Dagaaba leaders in March and April 2020. We supplement these with conversations held with seven gatekeepers and scholars of marriage practices across the two ethnicities in northern Ghana, namely, the Frafra (three) and Builsa (four) of north-eastern Ghana, in December 2021. Other important sources of data are the authors’ lived experiences of the cultural norms governing the bridewealth and brideservice (two Dagaaba men and a female Dagao by marriage). As bridewealth varies within and across ethnicities, our narratives are based on the specific congeries of the ethnicities under consideration.

The social worlds of the Dagaaba, similar to most of the ethnicities across Africa, are complexly interlocked with the worlds of ‘beings’ perceived to be other-than-human. These other-than-human ontologies include the spirits of the ancestors, witchcraft, magic/*juju* holders. Following the pervasiveness of these entities, the occurrence of good and bad events, including crop failure or inexplainable deaths, is believed to be punishment from the mystical ontologies (Akurugu & Degnen, 2022). Customarily, Dagaaba's marriage practices including the brideprice are closely bound with mystical forces. Prior to it being sent to the bride's agnatic kin, it is dedicated to the spirits of the male ancestors in a ceremony performed in the *kpeezaga/kpeedie*, ancestral room. As we argue below, this dedication ritual contributes to constraining women's control over their sexuality and productivity in the marriage.

The broader research from which data for this was drawn relied on feminist ethnographic epistemology. Feminist ethnography brings together such central elements of both feminism and ethnography as partnership, collaboration and empathy (Clifford & Marcus, 1986; Stacey, 1988; Skeggs, 2001). Feminist ethnography afforded the first author an opportunity to locate herself and the research participants in the same critical plane of knowledge generation. Consistent with the principles of feminist ethnography, the author participated in the daily lives of the research participants as well as attending such rituals as funeral and widowhood rites, preparations towards the marriage payment and travelling to markets together with the women. She also spent time at the settlement's market place with women who sell assorted soup ingredients. Discussions often covered such issues as the role of the bridewealth in exercising control over women's autonomy, the andro-centric ancestors and violence against women. The discussions yielded quite illuminating insights, which we draw on for analysis in the following section. Feminist ethnographic principles offered the author an opportunity to attend to the subtleties involved in the practice of bridewealth, drawing attention to its benefits and limitations while eschewing the imperialising and urban-biased valence of bridewealth feminism in the study settings (see Akurugu et al., 2021). Akurugu et al. deploy bridewealth feminism to designate feminist activism that is premised on undoing the institution of the marriage payment for the constraints it exercises over women's autonomy. Final ethics approval for the research was granted by the Faculty of Humanities and Social Sciences Ethics Committee at Newcastle University, England. In the ensuing section we discuss the findings of our research, relying primarily on data from the ethnography conducted among the Dagaaba of north-western Ghana and insights from key informant interviews across settings and ethnicities such as the Frafra and Builsa.

## The marriage payment in northern Ghana

**Figure 1.**
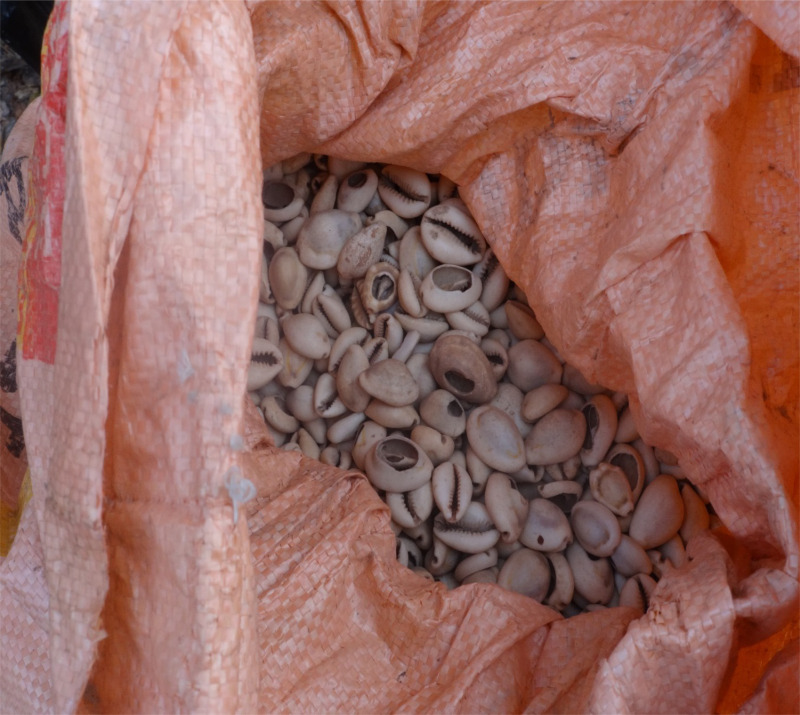
Cowrie shells in a sack ready to be taken to Papu, near Serekpere, as bridewealth.

The payment of bridewealth occupies an important position in the marriage process across ethnicities in northern Ghana, including Dagaaba, Frafra and Builsa nations. Among the Dagaaba, the bridewealth, *kyeuri*, mostly comprises such items as *libi*-*peԑle* (cowrie shells, see Figure 1), fowls, tobacco and cola nuts. Considerable flexibility characterises brideprice and different modalities govern various Dagaaba congeries. In some cases, the amount of cowrie shells must be commensurate with that which was paid for the bride's mother. In other instances, bonds of solidarity between the families or settlements of the bride and groom lead to a reduction in the payment, and if the bride's agnatic kin determine that in the past the groom's family charged a high brideprice for a marriage, they are likely to also insist on an exorbitant payment. Nonetheless, there are also standardised marriage payments across the Dagaaba settings. At present, an average of 2000 cowries, or cash equivalent, are generally accepted as bridewealth in Serekpere and its environs. This amount represents a grave reduction from an amount of 20,000 and above cowries that was accepted in the same area in the 1990s. Within the context of neoliberal economic policies, and dwindling agricultural productivity levels in this fragile ecological zone, our interaction with gatekeepers revealed that the cutback on the number of cowries is intended to make marriage affordable. This reduction in the bridewealth diverges from the extremely commercialised payments reported in urban areas in Ghana and elsewhere (Shope, 2006; Posel & Rudwick, 2013). Importantly, the payment is not contingent upon the circumstances of the bride, that is, whether the bride is highly educated, divorced, has already given birth or not as reported elsewhere (Borgerhoff Mulder, 1989, 1995; Anderson, 2007). The customary norms governing the practice of bridewealth are set out if negotiable irrespective of the bride's socio-economic status or the number of rights transferred.

The bridewealth among the Dagaaba, as across societies in rural northern Ghana, is a complex process of negotiations involving the agnatic kin of the bride and groom and, normally, a third party, called a *dendeo*, a marriage liaison. Normally, a marriage liaison is a man from the groom's village who has some relations in the bride's settlement. The *dendeo* leads the groom's family to the bride's agnatic kin, first to formally ask for her hand in marriage and to pay the brideprice. The relations of the *dendeo* with the bride's family mean that the latter is considerate to the groom and his kin. The former also pleads on behalf of the groom's kinsmen if the bride's family are seeking to charge an exorbitant brideprice. Once the bridewealth has been transferred to and accepted by the bride's kin, the marriage union is formed and legitimated.

Normatively, the *yirdandoo*, the head of the family, rather than the father of the bride, is the custodian of the cowries brought as marriage payments. At a group discussion in Nator, near Serekpere, demonstrating the role of the head of the clan, Mwinyella, a research participant, explained, pointing to his paternal uncle who is the head of his clan, that: ‘this man is my father and so when I marry it will be his duty to [pay the bridewealth] for me’. Yet, for families without resources designated as bridewealth, the groom and his agnatic kin are responsible for raising it. Anderson (2007: 159) reports that in settings across Africa, the marriage payment is normatively raised by the groom's ‘father, grandfather and father's brothers, and with mother's brothers making small contributions’. This differs, to an extent, from the Dagaaba account presented here. Yet it resonates with bridewealth practices among some Frafra congeries, where normatively the groom's father and father's brothers are responsible for paying the bridewealth and where he is unable to raise it the groom turns to his father's or mother's brothers for support.

### Dedication rituals and the transfer of marriage payment

Prior to the transfer of the cowries to the bride's agnatic kin, the male elders of the groom's family take a few of the cowries to the *kpeezaga*, mentioned above, where dedication sacrifices are performed to commit the prospective bride under the protection of the ancestors, and to ask for blessings, especially children for the marriage. The dedication ritual is performed before the marriage payment. Following the dedication, the cowries are transferred by male members of the groom, led by the *dendeo*, to the bride's agnatic kin, and once accepted, the marriage union is legitimately formed. It is, however, important to note the changing trend these days, that some brides and grooms live together and bear children before brideprice is paid. The dedication ritual has been referred to as the ‘ratification of the slavery of the woman’ by Dery (2013: 11) as it exercises great control over the bride's autonomy. Informed by inheritance arrangement, the bridewealth secures rights over the woman, her sexuality, labour and children for the man and his agnatic kin. The sacrifice prohibits the bride from having sex with another man; multiple mating is thus prohibited for the woman. Any woman who breaks this taboo is considered to have desecrated the marriage and has to be cleansed of the contagion.

Similarly, among the Frafra of north-eastern Ghana (the first author's natal ethnic identity), the marriage payment comprises mostly of four cows, a red cock called *nu-nua*, marriage fowl, a smock and local gin. The cows may be paid all in one go or in instalments. Among the Frafra, it is the payment of the red cock that legitimates the marriage. Once the fowl has been accepted, both families of the bride and groom recognise the marriage as a valid one. Yet before the fowl is accepted, in most cases some or all the cows will have to be paid. A Frafra bride who sleeps with another man, irrespective of the circumstance, is also believed to have defiled the marriage and thus requires purification. The payment exerts normative control over the bride's life.

Within the context of the Dagaaba, the dedication ritual and the subsequent payment exercise normative constraints on the woman. Similarly, among the Frafra people, the bridewealth together with cultural expectations of docility exercise enormous restrictions on the autonomy of women. Hence, the institution of the payment and the accompanying social expectations, rather than finalising the marriage payment as reported by Horne et al. (2013) or the number of rights transferred at marriage (Borgerhoff Mulder, 1995), constitute normative constraints to women in northern Ghana. Based on the constraints that the marriage payment exercises on women, some activists advocate the undoing of the institution (see Akurugu et al., 2021). However, all over the world women are oppressed in diverse ways. Indeed, even in settlements across Ghana where bridewealth is very small or not applicable, women are still oppressed. This is well illustrated by considering the Builsa people of northern Ghana.

Similar to other African ethnicities (see Borgerhoff Mulder, 1989), the marriage process of the Builsa is complex, involving several stages, payments and rituals. According to our research participants, bridewealth as described in the anthropological literature does not apply to Builsa marriage practices. Indeed, in an interview, one research participant explained that; ‘I wouldn't call them payments but rituals’. Yet two important rituals that legitimate a Builsa marriage are *akaayale le o boro, meaning* ‘seek not, she is there (with us)’ and the *nansuing ligka*, closing the gate (Adiita, 2011: 26). The *akaayale* comprises cola nuts, tobacco and a small amount of money for performing rituals. Wanitzek (1998) estimated the cash at 4 pesewas (less than $0.01). The *akaayale* follows the abduction of the bride, the customarily most common way of marrying. This ritual, often performed three to four days after the abduction, seals the marriage. The *nansuing ligka* rites finalise the marriage. During abduction, it is believed that the groom opened the bride's house entrance and the *nansuing ligka* symbolically brings a closure to it. It is made up of an animal – fowl, goat or sheep, a hoe blade and tobacco (Adiita, 2011). Among the Builsa, although women are in subjection to men, the ownership of the woman is not transferred to the husband and his kin as reported in the anthropological material (Lévi-Strauss, 1969). A research participant explained: ‘the rites do not give the husband and his family the right to own the woman’. Reacting to views among the Frafra and Dagaaba that the bridewealth is a driver of violence against women, the participant explained:

Women occupy a subordinate position, but […] she can move out if she is not pleased. Abusive behaviour by the man is not tolerated since the woman is always free to return to her father's house. If she goes [and] the man's family fails to follow up and apologise, the woman can choose to remarry and they have no right to accuse anyone.

The Builsa bride thus seems to have more autonomy as compared with others and our participants attribute this leverage to the absence of the bridewealth. Yet she occupies a subordinate position vis-à-vis her male folks. We also see the important role of the family/community in sustaining the marriage, which tends to be neglected in the discourses on bridewealth. Among the Builsa people, there is no marriage payment, although women's autonomy and control over decision-making power are no less limited as compared with men, and this is the case for many ethnicities across northern Ghana and across bridewealth-practising settings in Africa.

Within the context of the ethnicities under consideration here, the marriage payment completes the marriage process. The woman now becomes a legitimate member of the marital family; women whose bridewealth has not been paid are not considered properly married (see also Behrends, 2002; Dery & Akurugu, 2021). Subsequently, control over the woman is transferred from her agnatic kin to her husband's kin. The bride has no control over the products of her labour on the farm because they are *a dↄↄ kunkure boma*, proceeds of the man's hoe. This arrangement, the transfer of control and authority over women, has negative implications for women's autonomy; they are required to obtain permission from their ‘owners’ in all instances before taking any major decision. Those who do not comply with this normative requirement are thought of as behaving as though they *owned* themselves, submitting to no man's authority. In the Dagaaba settings such women are often called *pog gandaba* (singular-*pog gandoa*). Over the years, the meanings of *pog gandoa* have been contested. In one respect, it is interpreted to mean the antithesis of respectable femininity. A *pog gandoa* embodies assertive qualities (see Akurugu, 2020 for a detailed discussion). Nevertheless, the bride does not belong fully to her new family as a non-lineage member; her loyalty cannot be trusted since she might one day leave. She is, however, the *deԑ pↄge*, the woman of the house, in charge of the day-to-day management and funeral rituals in the community.

The bridewealth of the Dagaaba and Frafra departs from the practice of the Kipsigis of Kenya, where it is one-off and non-refundable (Borgerhoff Mulder, 1995). Secondly, it differs from that described by Horne et al. (2013), examined earlier. The focus of Horne et al. on the relationship between bridewealth and women's reproductive autonomy is restrictive and elides bigger issues such as the way in which the marriage payment exercises control over the entirety of a woman's life and creates subordinate positions for women in the marital family. Yet these scholars claim that ‘the theory is applicable to communities in which bridewealth is practiced’ (Horne et al. 2013: 508). The broad-based approach without references to specific bridewealth-practising settings is less useful. The discussion here shows that the marriage payment unites families and communities since it is a group rather than the individual couple's affair as reported elsewhere.

### ‘Woman is nin-baala, weak person’: bridewealth and constrained autonomy

**Figure 2.**
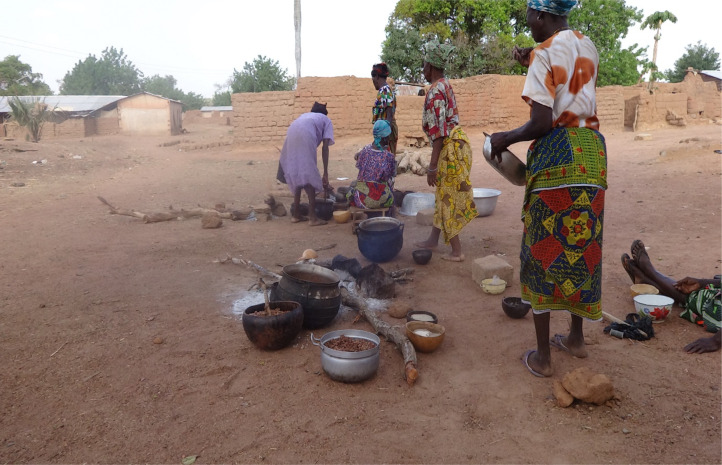
Daughters and wives of Serekpere, cooking ritual meals during final funeral rites.

Among the ethnicities under consideration, the bridewealth is an enduring institution. Customarily, it is impossible to complete the marriage payment as it spans over time and even on occasion outlives the marriage (see also Shope, 2006). In fact, according to most of the men we interviewed, one can never complete the marriage payment, as noted by an elderly participant in Serekpere: ‘you can never complete payment of the bridewealth’. This is because it is a bargaining process and even when a bride's kinsmen agree to her prospective husband's proposal of the number of cowries to pay for her, and accept it, they still remind them of an outstanding payment. This is to emphasise that the bride is worth more than any amount of money. Our research participants articulated the symbolic gesture of the bridewealth as a token to strengthen bonds between the two families (see Radcliffe-Brown & Forde, 1987; Kpiebaya, 1991). Kpiebaya (1991: 11), writing about the Dagaaba people, underscores that the payment is not an ‘outright purchase’ of the bride as her family retains the right to take her away from an abusive marriage. In addition to this, the bride does not lose all of her rights in the natal settlement; older daughters have ritual roles to play during funeral rites (see Figure 2). Nonetheless, in everyday discourse, women and men across the study areas refer to women in marriage as having been purchased, and thus being subordinate. For instance, a family head in Serekpere, pointing to his first wife, explained that:

for women of her age if the marriage is not working then it is her destiny; perhaps she hasn't been able to have children or she has only girls who have all married elsewhere. […] Where else can she go if the marriage is not working?

In his view, no man would be willing to return the bridewealth of such an old woman and this means that the payment bonds her perpetually to the marital family. This perspective of the clan head encapsulates the views of our research participants regarding the constraints of the marriage payment. The women often described a woman in marriage as a *nin-baala*, weak, person. In terms of women's social positioning relative to men, a women's group leader from the community explained that: ‘no money has been paid for a man in marriage and this is why a woman in marriage cannot compare herself to the men. The man is the owner of the house; he is a chief whereas the woman has been bought with the bridewealth.’ Another woman, a first wife who had been abandoned by her husband in the marital village, recounting the difficulties that staying in the marital family brings to her, explained that, ‘if you were not a *nin-baala* you would not leave your father's house and come to another person's house’. She concluded that ‘unless a woman recognises her *nin-baala* position and humbles herself she will not be able to live with a husband and his family’. The identity of the woman in marriage is often framed as *pↄge yԑŋ nin baala na* (‘woman is a weak person’). Thus, we can see the role of the marriage payment and the virilocal residential pattern in creating subordinate identities, and in constraining women's autonomy.

Despite the connection between bridewealth and constrained women's autonomy, the idea of abolishing the marriage payment was consistently rejected throughout our interviews across different ethnicities in northern Ghana. For our research participants, without the bridewealth there will be no marriage and the legitimacy of the bride and her children stand threatened in the marital family. Behrends (2002) and Akurugu et al. (2021) both highlight the challenges that ‘free’ marriage, marrying out brides without the bridewealth, engendered for the women and their children in north-western Ghana. The ‘free’ wives and their children were not recognised as legitimate members of the new family. Consequently, rather than unshackling the woman in marriage, ending the practice of bridewealth will further worsen her already vulnerable position. Across many contexts in Africa, the bridewealth is similarly revered, securing various kinds of entitlements and social status for women in marriage (Borgerhoff Mulder, 1995; Shope, 2006). Returning to the honest signalling theory discussed earlier, as the bridewealth and brideservice are culturally the responsibility of the groom's agnatic kin (although in recent times where families lack common pull resources designated for this purpose grooms contribute to it), signalling the communitarian valence of marriage in the study context, the payment provides information about the mate's kin as opposed to honestly signalling the suitability, potentials or otherwise of the groom. Indeed, within the context of this study, there is no customarily recognised marriage in the absence of the marriage payment and rituals. From analysis here, undoing the bridewealth is critically constrained as a means of liberating women from oppressive gender norms. In the concluding section below, we reflect on the potential of invoking the strong communitarian values as encapsulated by the Ubuntu philosophy and indigenous norms as important sites for negotiating women's rights in settings across Africa.

## Conclusion(s): implications of bridewealth for gender equality and advocacy

In this article, we have examined marriage practices and payment across settings in northern Ghana. In our concluding reflections, we explore the prospects of invoking an African philosophy and traditional institutions, including the traditional court system and Ubuntu, as useful means for negotiating women's rights and gender equality. Our analysis has highlighted the instrumental role of the payment in these settings in legitimating marriage and children born in the union. This resonates profoundly with much of the anthropological literature from bridewealth practising societies in Africa (Anderson, 2007; Apostolou, 2008; Horne et al., 2013). Yet bridewealth varies markedly across contexts, including among the three ethnicities under consideration in this study. Our findings reveal that bridewealth prices among the Frafra and the Dagaaba are relatively high, yet lower than the exorbitant prices among the Zulu of South Africa and the Kipsigis of Kenya. In bridewealth practising settings, the payment is perceived as the basis of women's oppression and normalised violence, complicating gender equality and women's empowerment endeavours (Dery & Bawa, 2019; Akurugu et al., 2021). Consequently, to liberate women from patriarchal domination and to engender greater parity, some advocates argue, it is necessary to undo the institution. Yet rural women who bear the burden of this practice are averse to this undoing project in recognition of the potential to worsen their already tenuous position. For some, the practice is ‘here to stay’ as Shope (2006: 4) reports. Even where it is not practised, as among the Builsa, women are still oppressed in many ways. In the context of the Kipsigis, Borgerhoff Mulder (1995: 574) reports that it is neoliberal commercialisation of maize production that has significantly disadvantaged women rather than the exorbitant marriage payment. The author reports that ‘Married women had relatively secure rights due primarily to the regionally predominant “house-property complex” system that provided women considerable autonomy in the use of their husbands’ property’. In this case marriage served as an enabler of autonomy and this corroborates Ogbu's (1978: 256) finding that bridewealth improves women's status. According to Ogbu, bridewealth enhances women's status inasmuch as it legitimates marriages. He explains that bridewealth secures ‘reciprocal rights and obligations’ for both the bride and groom, even as he concedes that women in African societies occupy subordinate social positions. The point is that a concatenation of factors rather than the marriage payment alone constrains women's autonomy. Thus, targeting the institution, which is already undergoing considerable transformation, may do little to liberate women. It is important to note that ethnographically grounded work on alternative institutions, such as early female marriage or polygynous marriage, has reached similar conclusions (see Schaffnit et al. (2021).

We suggest that it is useful to preserve this enduring institution, while at the same time protect women in bridewealth-practising societies from its constraints as well as those beyond such societies. We furthermore propose a return to communitarian, African-centred institutions, such as indigenous court systems, that were/are pervasive in rural settings across Africa (United Nations Office of the High Commissioner for Human Rights, 2016), including northern Ghana, but have been eroded by the onslaught of colonialism. The courts were important avenues for resolving general and marital disputes and a reinvigoration of this system will be an important means of negotiating gender equality for women across Africa. Although the composition of the court was mostly male dominated, and women's interests were not always served, we propose that a reinvigorated customary justice system should incorporate the concerns, voices and interests of women. This is possible when the queen mother institution is empowered to function as an important means of protecting women from violence. In the so-called stateless societies of northern Ghana, the queen mother institution is a recent creation, unlike in the matrilineal societies of southern Ghana, where the institution has been an important component of royalty since time immemorial (Yelpaala, 1983). Kojo Yelpaala (1983) challenges views held by earlier Western anthropologists studying Dagaaba societies that their settlements were stateless. Instead, Yelpaala argues that the construction of the notion of statelessness was the colonial administrators’ strategy to conquer the Dagaaba people (see also Hawkins, 2002). The queen mother, the occupant, is often considered by members of society as a woman of great repute who is installed in a public ceremony. The queen mother is usually the wife of the chief or a female in the royal family. She is in charge of mobilising women for community development and resolving matters affecting women within the jurisdiction of the chief. It is within the context of the important social roles of the queen mother towards women that we suggest the need to incorporate this institution into a revived customary justice system. Enacting such changes may bring its own challenges, which should be explored fully. Our broader point here is to advocate that, rather than abruptly abolishing practices disparaged by the foreign gaze of global health and gender activists, but revered by community members deemed in need of assistance, we must explore more culturally sensitive and tailored alternatives to empowering women

## Research transparency and reproducibility

Data for this article is available upon reasonable request.
