# Marriage, bridewealth and power: critical reflections on women's autonomy across settings in Africa – CORRIGENDUM

**DOI:** 10.1017/ehs.2022.37

**Published:** 2022-09-02

**Authors:** Constance Awinpoka Akurugu, Isaac Dery, Paul Bata Domanban

The authors apologise that upon publication of this article the author, the author Paul Bata Domanban's surnames were listed incorrectly as Paul Domanban Bata

The online version of this article has been updated.

## References

[ref1] Akurugu, C., Dery, I., & Domanban, P. (2022). Marriage, bridewealth and power: Critical reflections on women's autonomy across settings in Africa. Evolutionary Human Sciences, 4, E30. doi:10.1017/ehs.2022.27 PMC10426024 37588918

